# The role of Aha1 in cancer and neurodegeneration

**DOI:** 10.3389/fnmol.2024.1509280

**Published:** 2024-12-24

**Authors:** Brian S.J. Blagg, Kevin C. Catalfano

**Affiliations:** Department of Chemistry and Biochemistry, University of Notre Dame, Notre Dame, IN, United States

**Keywords:** Hsp90, Aha1, cancer, tauopathy, protein–protein interaction, small molecule

## Abstract

The 90 kDa Heat shock protein (Hsp90) is a family of ubiquitously expressed molecular chaperones responsible for the stabilization and maturation of >400 client proteins. Hsp90 exhibits dramatic conformational changes to accomplish this, which are regulated by partner proteins termed co-chaperones. One of these co-chaperones is called the activator or Hsp90 ATPase activity homolog 1 (Aha1) and is the most potent accelerator of Hsp90 ATPase activity. In conditions where Aha1 levels are dysregulated including cystic fibrosis, cancer and neurodegeneration, Hsp90 mediated client maturation is disrupted. Accumulating evidence has demonstrated that many disease states exhibit large hetero-protein complexes with Hsp90 as the center. Many of these include Aha1, where increased Aha1 levels drive disease states forward. One strategy to block these effects is to design small molecule disruptors of the Hsp90/Aha1 complex. Studies have demonstrated that current Hsp90/Aha1 small molecule disruptors are effective in both models for cancer and neurodegeration.

## Graphical abstract

Hsp90/Aha1 is associated with increased Hsp90 ATPase activity and associated client maturation drives cancer and neurodegenerative diseases. Disrupting Hsp90/Aha1 is a viable strategy to treat these diseases.

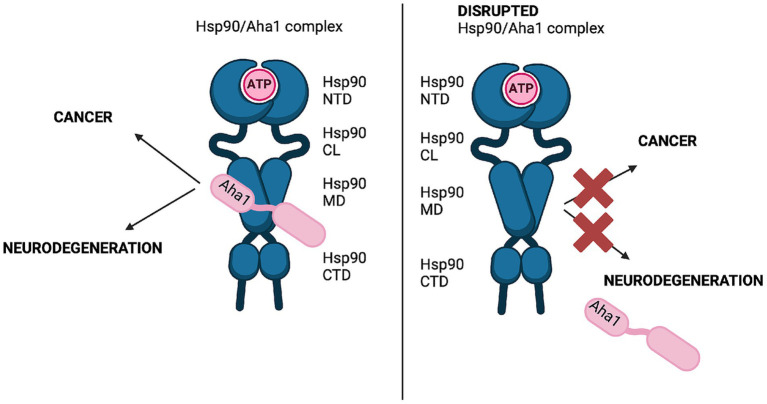

## 1 Hsp90 co-chaperone complexes: an introduction

**Figure 1:**
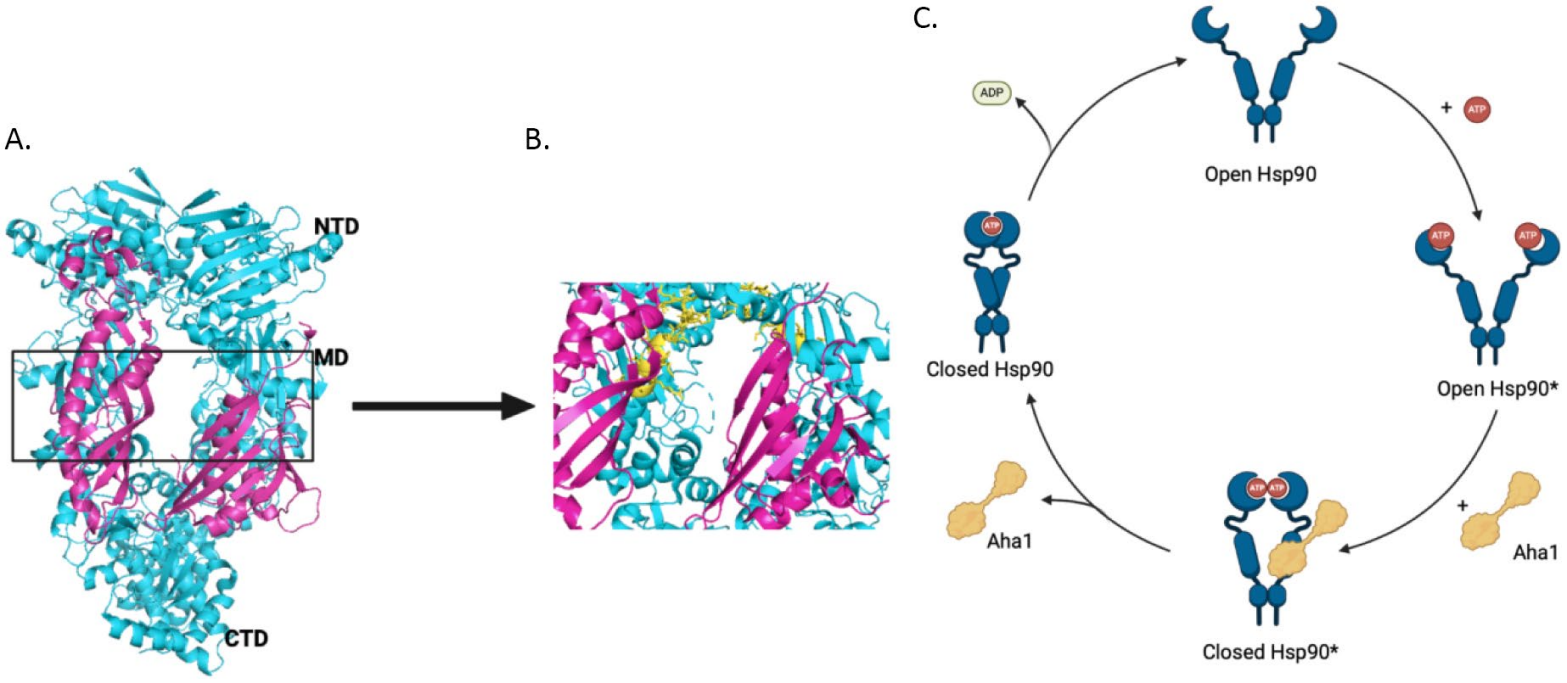
**(A)** The structure of Hsc82, the *S. cerevisiae* Hsp90 homolog in cyan and Aha1 in magenta are shown above with the Hsp90 NTD, MD and CTD labeled are shown for one protomer PDB 6XLF. **(B)** The MD of Hsp90 (cyan) with Aha1 (magenta) are shown with the Hsp90 catalytic loop (yellow), residues 370–390. **(C)** The unidirectional model of the Hsp90 chaperone cycle is depicted where co-chaperones like Aha1 drive the cycle, ATP hydrolysis and client maturation forward.

The 90 kDa heat shock protein (Hsp90) family accounts for 1–2% of the total protein in healthy cells and upwards of 4–6% in stressed/transformed cells. This family of molecular chaperones plays a vital role in proteostasis because they are responsible for the folding and conformational maturation of >400 client proteins. Many of these clients are dysregulated in disease states such as cancer, cystic fibrosis and neurodegeneration (Ihrig and Obermann, 2017; Koulov et al., 2010; Moll et al., 2022; Heider et al., 2021). To facilitate folding, Hsp90 must undergo large conformational shifts that are mediated by ATP binding and hydrolysis. These conformational changes lead to a closed complex, wherein the Hsp90 middle domain (MD) and N-terminal domains (NTDs) are dimerized (Figure 1A). These conformational shifts are regulated by partner proteins that bind various sites on Hsp90 termed co-chaperones (Hessling et al., 2009; Shiau et al., 2006; Richter et al., 2001; Jahn et al., 2014). One of these co-chaperones, called the Activator of Hsp90 ATPase activity homolog 1 (Aha1), binds the middle domain of Hsp90 and accelerates Hsp90 ATPase activity (Figure 1A). Aha1 is believed to accelerate Hsp90 ATP hydrolysis through interactions with the Hsp90 catalytic loop (residues 370–390) (Figure 1B). ATP hydrolysis and subsequent ADP release allows the folded client to dissociate from the Hsp90 complex (Li et al., 2013). The physiological importance played by Hsp90 led to the development of small molecule inhibitors of the N-terminal ATP binding pocket. Unfortunately, most of these molecules failed in clinical trials due to on-target toxicities (Peterson et al., 2012). In fact, they bind the NTD of all 4 Hsp90 isoforms (Hsp90α, Hsp90β, Grp94 and TRAP1) with similar affinity, which leads to the degradation of >400 clients and toxicity (Peterson et al., 2012). Some of these clients play important physiological roles and Hsp90 pan-inhibition was shown to induce cardio-, dose-limiting- and/or ocular-toxicities (Kim et al., 2009). One strategy to mitigate these toxicities is to target protein–protein interactions between Hsp90 and the co-chaperones that regulate the chaperone cycle. Such a strategy would allow Hsp90 to fold essential client proteins, while mitigating disease states induced by co-chaperone imbalances.

Disruption of protein–protein interactions via small molecules to modulate Hsp90 activity is garnering interest. For example, Hsp90 and the co-chaperone, Cell Division Cycle 37 (Cdc37), have been found prevalent in various cancers and neurodegeneration (Moll et al., 2022; Hurtado-Lorenzo and Anand, 2008; de et al., 2005). In fact, ~60% of the human kinome is dependent upon this heteroprotein complex for maturation and stability (Taipale et al., 2012). Disruption of this interaction results in the degradation of various kinase clients, some of which drive cancer growth and invasion. Hsp90 clients in the brain are known to play important roles in the phosphorylation of Tau species that accelerate neurodegeneration and are reliant upon Aha1 and Hsp90. The Hsp90/Aha1 complex presents an interesting and novel target because in unstressed cells, Hsp90 exists primarily as the free dimer. However, in stressed or transformed cells, Hsp90 exists in heteroprotein complexes, some of which include Aha1. These heteroprotein complexes drive disease states through Hsp90 activity and disrupting these complexes is an innovative treatment strategy, while avoiding deleterious effects in healthy tissue (Chiosis et al., 2023). This review details recent advances regarding the role played by Aha1 in the context of cancer, neurodegeneration and the development of small molecules that disrupt the Hsp90/Aha1 complex as a novel treatment strategy.

## 2 Regulation of Hsp90 by Aha1 and competing co-chaperones

As mentioned, Hsp90 undergoes major conformational changes to bind, fold and release >400 client proteins (Jahn et al., 2014). Human Hsp90 exhibits poor ATPase activity of 0.1 ATP/min, but is accelerated 4-fold by the co-chaperone Aha1 (Tripathi et al., 2014; Panaretou et al., 2002). The yeast co-chaperones, stress inducible phosphoprotein 1 (Sti1) and Aha1, were studied to better understand the role co-chaperones play in Hsp90 conformational dynamics. While Sti1 blocked ATPase activity, yeast Aha1 (yAha1) accelerated conformational changes even in the absence of ATP, which was likely accomplished by the accumulation of late intermediates (Figure 1C) (Hessling et al., 2009; Genest et al., 2013). These results suggest that conformational dynamics are controlled by nucleotide binding and release. Structural data from an x-ray crystal structure of apo-Hsp90 from *E. coli* in the absence of nucleotide displays an open conformation. However, in the presence of ADP, hydrophobic residues cluster together following conformational changes (Shiau et al., 2006). Furthermore, a recent study utilizing E33A mutants of Hsp90, wherein Hsp90 can bind ATP, but cannot hydrolyze the gamma phosphate, highlighted the importance of nucleotide binding and release. Several mutant Hsp90 orthologs with the mutation analogous to E33A in yHsp90 supported yeast growth, despite hindered ATP hydrolysis these mutants underwent conformational shifts, which suggested the binding and dissociation of nucleotide drives conformational shifts rather than energy from hydrolysis (Reidy et al., 2023). Hsp90 ATPase activity is significantly inhibited by ADP binding, however this is mitigated by Aha1 binding, suggesting that conformational dynamics driven by Aha1 are important for driving the cycle forward, including ADP release (Halpin and Street, 2017). Early in the chaperone cycle, the NTDs interact with the charged linker (CL) to limit rotational flexibility. However, the NTDs dissociate from the CL to enable NTD dimerization. Aha1 may accelerate this conformational change (Jahn et al., 2014). For example, when the Hsp90 CL sequence was replaced with highly flexible GGS repeats, Aha1 was unable to accelerate ATPase activity. This was attributed to difficulty dimerizing the NTDs, which emphasizes the role played by the NTD and the CL to limit rotation, but also the role Aha1 plays to regulate this interaction.

Aha1 was demonstrated to be a critical regulator of Hsp90 conformational changes, which led to kinetic studies focused on Hsp90 ATPase dynamics. Hsp90 ATP binding was simulated upon asymmetric binding of the non-hydrolyzable ATP analog, AMP-PNP, along with ATP cooperative binding. Conversely, the addition of 10 μM Aha1 increased nucleotide residence time, which suggests Aha1 accelerates movement to the closed state even in the absence of ATP (Wortmann et al., 2017). Additional experiments demonstrated that yAha1 imparted thermotolerance in yeast under Hsp90 depleted conditions, which indicates that Aha1 is a vital component of the Hsp90 chaperone machinery (Lotz et al., 2003). Further experiments examined a diverse array of human tumors, which exhibit the presence of Hsp90 chaperone complexes. Human Aha1 (hAha1) was found to be a vital component of these complexes and depletion of both Hsp90α and Aha1 led to cell death. Further, they demonstrated that Aha1 was necessary for the acceleration of Hsp90 dependent client maturation, further highlighting that Aha1 is a vital member of the chaperone machinery and may be a therapeutic target to control proteostasis (Rodina et al., 2016).

Aha1 is ubiquitously expressed among eukaryotic organisms and exhibits high sequence identity among metazoan eukaryotes at the conserved RKxK motif, residues 75–78 in hAha1, which is vital for interactions with the catalytic loop of Hsp90 (Figure 1B). Mutation of this motif impairs Aha1 stimulated Hsp90 ATPase activity (Horvat et al., 2014). However, among the Aha1 orthologs from yeast to higher eukaryotes, a NxNNWHW motif, residues 23–27 in hAha1 is also highly conserved. Experiments demonstrated that this motif extends toward the Hsp90 NTDs in Hsp90/Aha1 complexes. Deleting this sequence increased nucleotide dissociation from Hsp90, which is important for directionality of the Hsp90 protein folding cycle. Solution NMR experiments revealed that the yAha1 NTD residues adjacent to the NxNNWHW motif interact primarily with the Hsp90 MD. Interestingly, the yAha1 CTD stabilizes the Hsp90 NTDs and interacts with K178 on Hsp90, which is SUMOylated to recruit Aha1 (Mollapour et al., 2014; Wolmarans et al., 2016). LaPointe and collogues proposed that initial interactions between Aha1 and the Hsp90 NTDs facilitate conformational changes in Hsp90, whereas interactions with the Aha1 CTD stimulates ATPase activity (Wolmarans et al., 2016).

**Figure 2:**
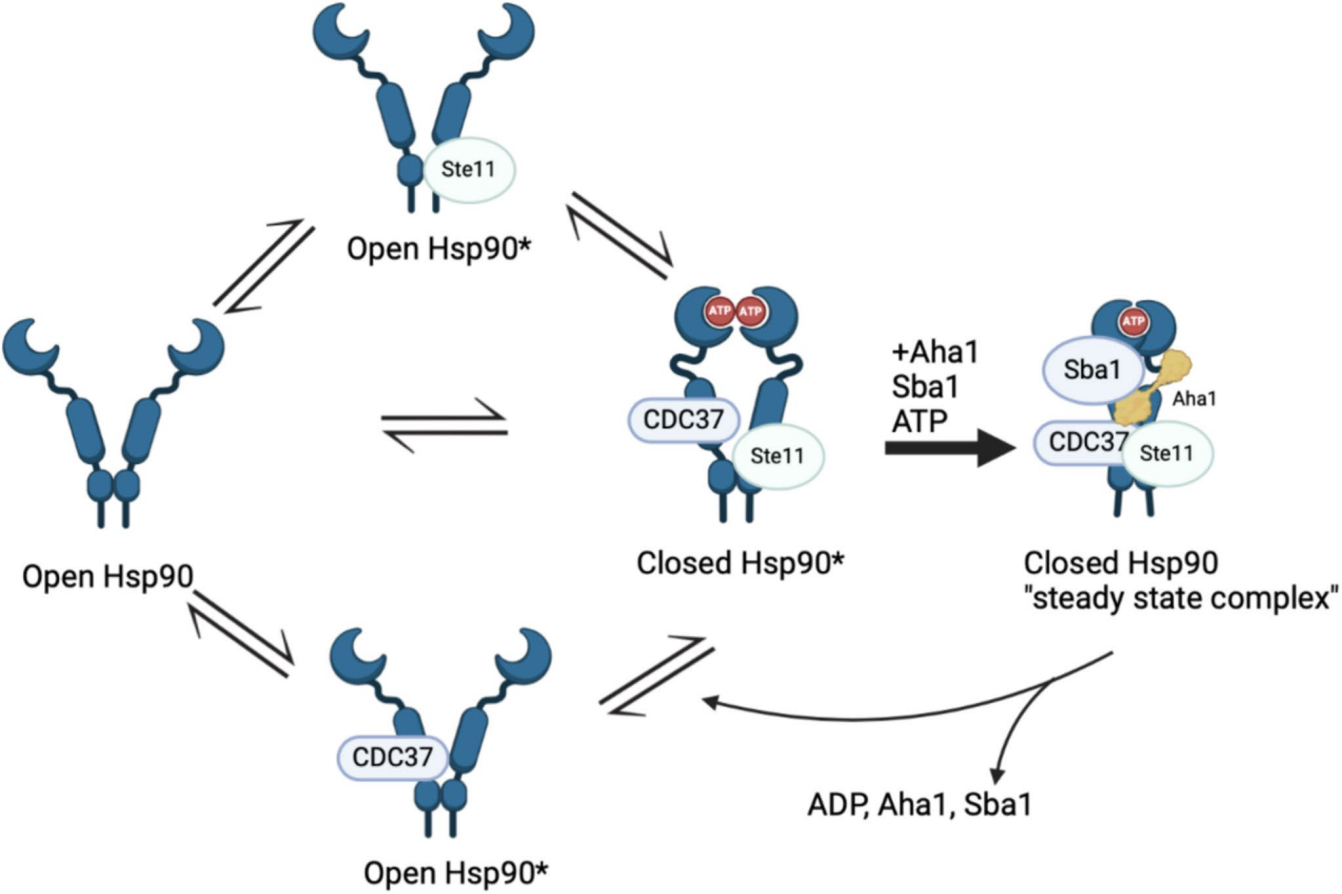
Recent Hsp90 chaperone model depicting four states in a population model, Hsp90 open, short lived Hsp90 open, short lived Hsp90 closed and Hsp90 closed. The initial three states are in “delicate balance,” where introducing ATP, Sba1 and Aha1 significantly increased ATP hydrolysis that is tied to client maturation. The final state includes all five components and is removed from the cycle in “steady state.”

Recent work by Hugel and coworkers via single molecule Förster Resonance Energy Transfer (smFRET) studies expanded the Hsp90/Aha1 model (Vollmar et al., 2024). They demonstrated that Hsp90 exists in four populations, which includes an open state, a short-lived open state, a short-lived partially closed state and a closed state (Figure 2). yAha1 binding led to a greater population of the closed state (Wortmann et al., 2017; Schmid and Hugel, 2020). They showed that yHsp90 required a kinase client (Ste11), yCdc37, yAha1, Sba1 and ATP to assemble a “steady state complex” that exhibited the greatest ATPase activity (Figure 2). They proposed that the role played by ATP binding to the Hsp90 NTD is to stabilize the steady state complex. The proposed mechanism exhibited distinct differences when compared to the unidirectional chaperone cycle shown in Figure 1 and suggests that it is the co-chaperones that dictate direction of the folding cycle (Chiosis et al., 2023; Mondol et al., 2023). Furthermore, it is likely that interactions with different co-chaperones direct Hsp90 to interact with specific clients.

**Figure 3:**
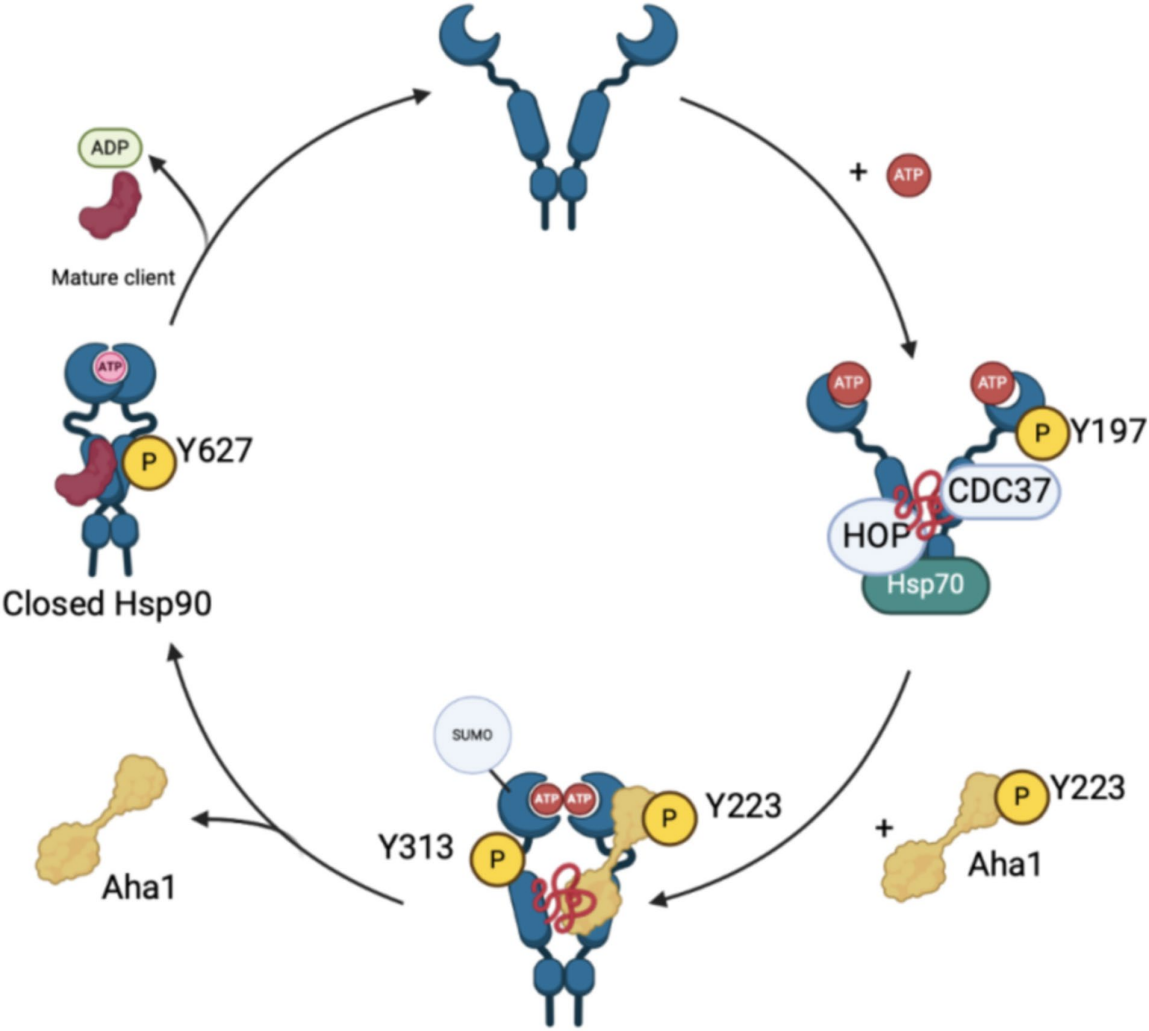
The recruitment and displacement of various co-chaperones that move the Hsp90 cycle forward depends upon post-translational modifications that enhance or hinder these interactions as shown in the figure above.

Although the biophysical data discussed above emphasizes the dynamic nature of co-chaperone binding to Hsp90, it does not explain how Aha1 interacts with Hsp90 and how it facilitates Hsp90 ATPase activity. Recent studies have emphasized the importance of posttranslational modifications (PTMs) to regulate protein–protein interactions. For example, Aha1 is expressed at substoicheometric concentrations as compared to Hsp90 and exhibits micromolar affinity for Hsp90. Not surprisingly, PTMs regulate Hsp90/Aha1 interactions to enhance or disrupt complex formation. A modification that enhances Hsp90/Aha1 interactions is the asymmetric SUMOylation of Hsp90 K178 in yeast and K191 in human Hsp90 following ATP binding (Figure 3). In contrast, mutation of this lysine prevented SUMOylation and Hsp90-Aha1 binding. Furthermore, mutant yeast with hindered Hsp90/Aha1 interactions displayed reduced sensitivity to N-terminal domain (NTD) Hsp90 inhibitors and did not elicit the pro-survival heat shock response, which suggests Aha1 holds Hsp90 in a conformation with greater affinity for inhibitors. Studies have shown that oncogenic transformation of mouse fibroblasts enhances Hsp90 SUMOylation, Aha1 binding and hypersensitivity to Hsp90 pan-inhibitors, which suggests that both inhibitors and Aha1 prefer the same Hsp90 conformation (Mollapour et al., 2014). Lysine 594 in yeast is important for facilitating the closed state of Hsp90 and mutation of this residue allows Aha1 binding, but hinders ATPase activity. Similarly, Hsp90 client maturation is also hampered by this mutation (Rehn et al., 2020). These data provide a biochemical basis for a mechanism by which Aha1 stimulates ATPase activity by facilitating transition to the Hsp90 closed state to drive client maturation.

Phosphorylation of residues on both Hsp90 and Aha1 also regulate protein–protein interactions. Experiments by the Mollapour group demonstrated that Y223 on Aha1 is phosphorylated by c-Abl kinase, which enhances the affinity of Aha1 for Hsp90α and leads to greater ATPase stimulation (Panaretou et al., 2002; Gonfloni, 2014; Wolfgeher et al., 2015). Furthermore, Aha1 phosphorylation at Y223 led to Aha1 polyubiquitinylation and proteasome-mediated degradation (Dunn et al., 2015). The degradation of Aha1 represents a negative feedback loop that regulates Hsp90/Aha1 interactions. Phosphorylation of Y313 Hsp90 enhanced Aha1 binding 3.5-fold, which can be increased by molybdate. Following Hsp90 phosphorylation, Aha1 displaces HOP, and facilitates progression through the Hsp90 ATPase cycle (Xu et al., 2019). Subsequent phosphorylation of Hsp90 Y627 displaced Aha1 to reset the cycle (Figure 3) (Xu et al., 2012). Regulation of Aha1 binding via phosphorylation is important as Hsp90/Aha1 facilitates the maturation of some clients, but is deleterious for others. For example, enhancing the Hsp90/Aha1 interaction through PTMs decreases the stability of cystic fibrosis transmembrane conductance regulator (CFTR) and glucocorticoid receptor, but enhances the maturation of some kinases (Mollapour et al., 2014; Dunn et al., 2015). Stability of CFTR was compromised by the Hsp90/Aha1 complex, however Aha1 depletion led to the stabilization of CFTR (Wang et al., 2006).

The biochemical data demonstrate that disruption of the Hsp90/Aha1 complex can be beneficial to treat various diseases. Interruption of the Hsp90/Aha1 complex can be assisted by structural data via analysis of protein–protein interactions using X-ray crystallography, SAXS, solution NMR structure analysis and/or single particle cryo-EM. Prodomou et al. solved the first structure of the Hsp90/Aha1 complex via X-ray crystallography using the yeast Hsp90 homodimer and the Aha1 NTD (residues 1–153). Aha1 was truncated because isothermal titration calorimetry demonstrated that the Aha1 NTD is responsible for Hsp90 ATPase stimulation and the flexibility of full length Aha1 presented additional crystallography challenges (Meyer et al., 2004). Overall, the data demonstrated that the Aha1 NTD facilitates repositioning of Hsp90 R380, which interacts with the gamma phosphate of ATP to catalyze ATPase activity. Not surprisingly, when Aha1 was absent, ATPase activity is significantly diminished (Horvat et al., 2014; Meyer et al., 2004).

Although experiments have clearly demonstrated that the Aha1 NTD is vital for enhancing Hsp90 ATPase activity, the role played by the Aha1 CTD is less studied. Molecular dynamics studies were performed to better understand the Aha1 CTD and suggested that full length Aha1 pushes the Hsp90 conformational equilibrium toward the NTD dimerized ATP hydrolysis competent closed state. Solution NMR experiments demonstrated that Aha1 interacts with Hsp90 in a sequential manner that promotes Hsp90 NTD dimerization via an asymmetric mechanism (Retzlaff et al., 2010; Blacklock and Verkhivker, 2013). Furthermore, studies have shown that the Aha1 NTD interacts with the Hsp90 MD to facilitate NTD dimerization, while the Aha1 CTD interacts with the Hsp90 NTD. Initial Aha1 CTD interactions appear to trap ATP in the nucleotide binding pocket and subsequently facilitate hydrolysis/nucleotide exchange. Hsp90 ATPase activity and conformational shifts are influenced by interactions with the Aha1 CTD, but initial binding of the Aha1 NTD is required (Oroz et al., 2019). Structural details were elucidated via single particle cryo-EM, wherein the data supported a multistep mechanism for Aha1 stimulation of Hsp90. Upon Aha1 NTD binding to Hsp90, the CTD facilitates Hsp90 NTD dimerization and rotation about the charged linker to promote ATP hydrolysis. This conformation exhibits enhanced binding with a conserved N-terminal motif on Aha1 (Mercier et al., 2019; Liu et al., 2020). After which, the Aha1 NTD rotates to bind the Hsp90 NTDs and stabilize dimer formation and asymmetric ATP hydrolysis followed by Aha1 release (Liu et al., 2020). Collectively, these data demonstrate that the Aha1 NTD enhances the hydrolysis of ATP and the Aha1 CTD accelerates Hsp90 conformational dynamics.

Additional evidence has shown that co-chaperones regulate Hsp90 conformational dynamics and alter various protein substrates. For example, one study examined a set of human patient tumors whose growth was fueled by high molecular weight Hsp90 complexes. In most of these tumors, Aha1 is a critical component of these complexes and is vital for the maturation of Hsp90 clients such as EGFR and p-S6K. In addition, Aha1 enhances sensitivity to Hsp90 inhibitors (Rodina et al., 2016; Dunn et al., 2015). Experiments found that Hsp90/Aha1 specific clients include RNA splicing machinery and DNA repair machinery (Sun et al., 2012). For example, the Hsp90/Aha1 complex is required for the maturation of Dicer1 in U2OS osteosarcoma cells, which suggests that Aha1 is responsible for the maturation of microRNA. Upon Dicer1 depletion, mutant E67K Aha1, which does not bind Hsp90, partially rescued Dicer1 levels. However, deletion of the initial 20 amino acids in Aha1 prevented rescue of Dicer1. Dicer1 is at least partially chaperoned by Aha1 independent of Hsp90 and is dependent on the first 20 amino acids in Aha1 (Liu et al., 2022). In addition to RNA metabolism, Aha1 is associated with DNA repair. For example, one study demonstrated an association between Aha1 and the DNA double strand break repair protein, Rad51, in *S. cerevisiae* yeast. Interactions between Hsp90 and Rad51 are dependent upon Aha1 association with Hsp90, which also enhances the stability of Rad51. Aha1 also translocates Hsp90 to the nucleus in response to DNA damage, wherein Aha1 accumulates (Fangaria et al., 2022; Rani et al., 2024).

Aha1 chaperones clients beyond those that regulate DNA repair and RNA metabolism. Interestingly, Aha1 has no ATP-binding site nor ATPase activity and does not appear to cooperate with other chaperones to facilitate client folding since Aha1 is unable to release these proteins, resulting in holdase activity. Despite the lack of an active folding cycle, Aha1 can facilitate ubiquitinylation by the recruitment of CHIP (carboxyl terminus of Hsc70-interacting protein) to stimulate proteasome-mediated degradation via interactions with Hsp90 (Tripathi et al., 2014; Tang et al., 2023). More active roles for Aha1 were demonstrated via the maturation of the sulfate transferase, SULT1A1. SULT1A1 is a protein that transfers sulfates to metabolites for excretion via urine. The maturation of SULT1A1 is independent of Hsp90, but Aha1 is necessary (Liu and Wang, 2022). Interestingly, chaperone and holdase activity manifested by Aha1 is heavily associated with the N terminal fragment of Aha1. In fact, residues 1–20 on Aha1 are important for chaperone activity, but the role of these 20 residues and their interaction with Hsp90 is poorly understood. LaPointe and colleagues demonstrated that deletion of these residues (Aha1Δ20) enhances Hsp90 ATPase stimulation *in vitro* with purified recombinant Hsp90. However, deleting the first 27 amino acids (Aha1Δ27) reduced Hsp90 ATPase stimulation comparable to full length Aha1. Further mutation of the Aha1Δ20 deletion mutant at residues 25–27, wherein WHW was exchanged for AAA, significantly decreased stimulation of Hsp90 ATPase activity and affinity for Hsp90α and Hsp90β. Surprisingly, both Aha1Δ20 and Aha1Δ27 transfected MDA-MB-231 cells exhibited significantly lower interactions between mutant Aha1 and Hsp90. Both conditions also showed a loss of glucocorticoid receptor (GR) interaction with Aha1 via Co-IP. Furthermore, both Aha1Δ20 and the Aha1 E67K exhibited a loss of interaction with Hsp90 and GR, which demonstrated an important role for the initial 20 residues of Aha1 (Hussein et al., 2024).

Several disease states including cancer and neurodegenerative disease manifest chaperone imbalances and other Hsp90 interactors must be able to displace Aha1 for proper chaperone function. Several examples of competition befall various co-chaperones. For example, extracellular Aha1 interacts with Hsp90, but can be displaced by the inhibitory co-chaperone TIMP2. Increased interaction between extracellular Hsp90 and Aha1 facilitates activation of matrix metalloproteinase-2 (MMP-2), which increases cancer invasion/metastasis. TIMP2 mediated displacement of Aha1 stabilizes the ATP bound state of Hsp90, which reduces the activation of MMP-2 and consequently invasion/migration (Baker-Williams et al., 2019). Similar results were observed in experiments that focused on tuberous sclerosis complex 2 (Tsc1), which is another inhibitory co-chaperone of Hsp90. Tsc1 displaces Aha1 from Hsp90 and interacts with Cdc37 and protein phosphatase 5 (PP5), however phosphorylated Y223 Aha1 can displace Tsc1. Tsc1 increases the stability of kinase and non-kinase clients as well as CFTR, which contradicts Aha1 activity (Koulov et al., 2010; Woodford et al., 2017). Another co-chaperone that exhibits activity like Tsc1 was identified through investigation of immunommodulatory drugs including thalidomide. Thalidomide binds cereblon (CRBN), which chaperones CD147 to form a mature CD147-MCT1 complex that drives cancer progression. CRBN depleted cells accumulate membrane proteins in subcellular compartments. Interestingly, CRBN immunoprecipitates with Hsp90 and Aha1, wherein interactions between CRBN and Hsp90 reduce Hsp90 ATPase activity and increase holdase activity. The CRBN-Hsp90-Aha1 interaction is associated with increased transporter cell surface localization and enhanced stability. Understanding which moieties drive CRBN binding with Hsp90 and Aha1 could represent an attractive approach for the development of novel therapies for multiple myeloma or other conditions driven by membrane proteins (Heider et al., 2021; Eichner et al., 2016).

## 3 The role of Aha1 in cancer

Multiple cancers are dependent upon Hsp90/Aha1 for the maturation of client proteins that drive proliferation, migration and/or metastasis. Aha1 is associated with a variety of cancers including osteosarcoma (OS), hepatocellular carcinoma (HCC), colorectal cancer (CRC) and multiple myeloma (MM). Early studies indicated Aha1 is overexpressed in kidney and bladder tumor tissue when compared to normal tissue (McDowell et al., 2009). Moreover, analysis of 33 tumors via the cancer genome atlas revealed AHSA1, the gene encoding Aha1, RNA expression was elevated in breast invasive carcinoma, colon adenocarcinoma, lung adenocarcinoma, cholangiocarcinoma and prostate adenocarcinoma. Elevated Aha1 levels are correlated with increased HSP90AA1 expression in several cancers and decreased survival. Aha1 expression has also been shown to be an independent prognostic factor in other cancers (Li and Liu, 2022).

### 3.1 Osteosarcoma

**Figure 4:**
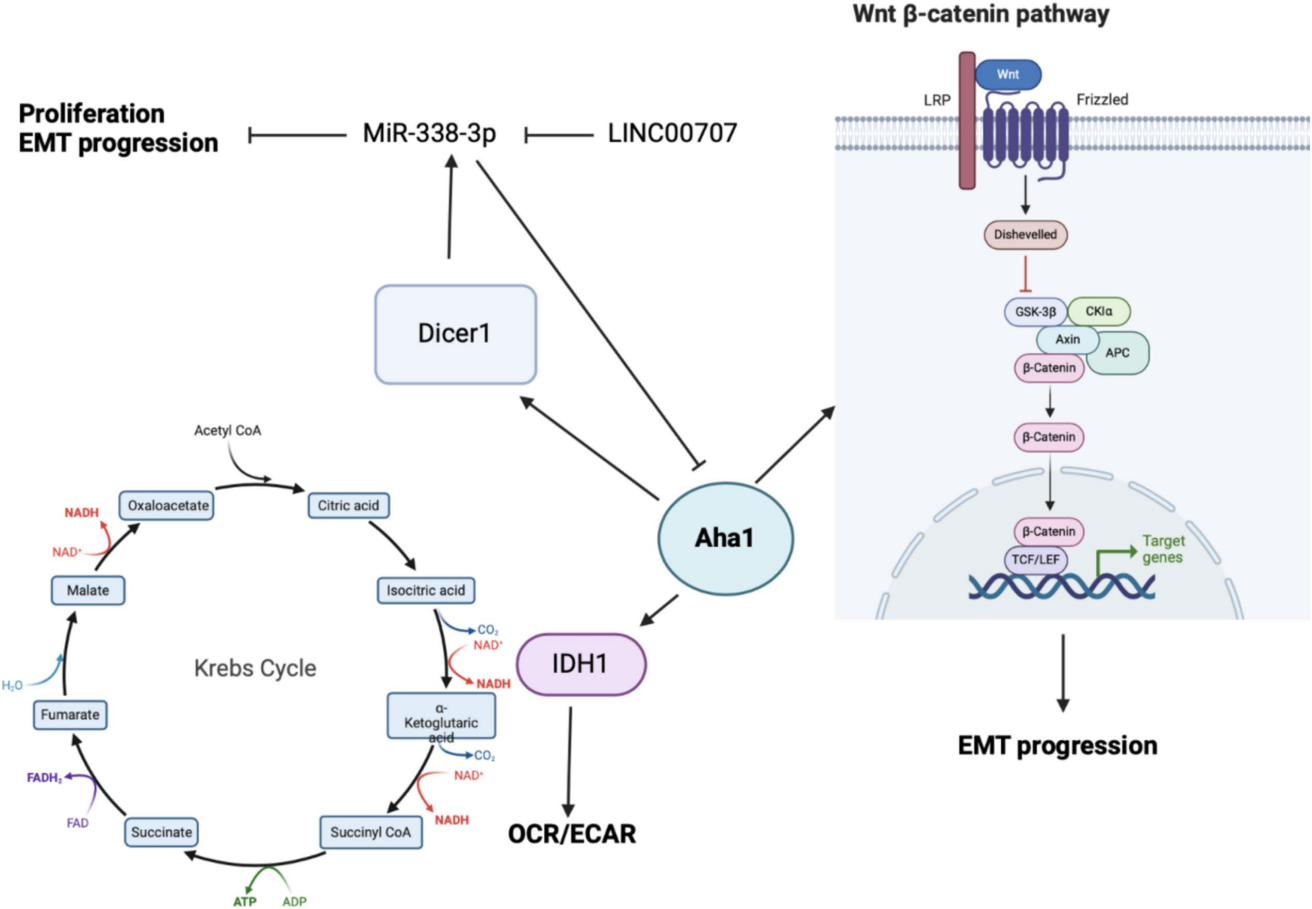
Cartoon depicting the mechanisms by which Aha1 overexpression mediates increased proliferation, invasion, EMT progression and subsequent metastasis in osteosarcoma.

Bioinformatic analysis of human cancers indicated elevated Aha1 expression in OS. OS is primarily found in adolescents and is associated with poor survival. Not surprisingly, OS along with elevated Aha1 expression correlates directly with poor patient outcomes (Zheng et al., 2021). Further studies that examine SaOS-2, IOR/OS9 and U2OS cell lines derived from human OS patients showed Aha1 overexpression to be increased by 12-, 24- and 23-fold as compared to osteoblasts (Guo et al., 2007). Subsequent experiments showed increased Aha1 mRNA transcription in MG-64, Saos2, MNNG/HOS, U2R, 143B, and ZOSM cells when compared to human osteoblasts (Zheng et al., 2021; Shao et al., 2016). Aha1 knockdown in these cells led to anti-proliferative activity, reduced migration, and a pro-apoptotic phenotype. Western blot analysis suggested that Aha1 knockdown significantly downregulated the Wnt/β-catenin pathway, while GSK3β and axin-2, which are negative regulators of this pathway, were increased (Figure 4) (Shao et al., 2016).

When Aha1 was knocked down in OS and methotrexate resistant U2R cells, they exhibited significantly decreased proliferation and migration. In contrast, when Aha1 was overexpressed, the cells exhibited increased proliferation and migration. Introduction of exogenous Aha1 to Aha1 depleted cells rescued these oncogenic characteristics, which indicate this effect is specific to Aha1 (Zheng et al., 2021). OS *in vivo* studies recapitulated *in cellulo* results, wherein BALB/c mice received tail vein injections with U2R cells transfected with Aha1 knock down or scrambled siRNA (siScr). Mice injected with the siScr cells exhibited significantly elevated tumor burden. Immunohistochemical analysis of tumors from siScr mice showed elevated proliferation markers in tumor tissue as compared to the Aha1 knockdown group. Similar to *in vitro* studies, mice with Aha1 knockdown tumors manifested decreased metastasis (Zheng et al., 2021). Collectively, these data show that Aha1 supports increased tumor growth and metastasis in OS.

While the biochemical data demonstrate that Aha1 mediates proliferation and metastasis in OS, the mechanism(s) by which this occurs remains unclear. One mechanism identified via seahorse experiments demonstrated that Aha1 knockdown in U2R cells decreased the extracellular acidification rate/oxygen consumption rate ratio (ECAR/OCR) (Han et al., 2020). However, the overexpression of Aha1 in U2R cells increased the ECAR/OCR, which suggests that an imbalance in Aha1 levels led to metabolic reprograming in OS. Moreover, Aha1 knockdown decreased glucose consumption and lactate production (Han et al., 2020). However, Aha1 overexpression led to greater ATP consumption and glutathione production, which favors biomass production and suggests that Aha1 is a regulator of energy metabolism. Analysis of metabolic enzymes indicated that isocitrate dehydrogenase 1 (IDH1) was significantly decreased in Aha1 knockdown cells and IDH1 was significantly elevated under Aha1 overexpression conditions (Han et al., 2020). Human OS tissue also manifests increased IDH1 and Aha1. Interestingly, IDH1 is expressed in the cytosol and peroxisomes, but not in mitochondria. IDH1 catalyzes the conversion of isocitrate to alpha-ketoglutarate resulting in NAPDH production. This activity is associated with histone demethylation and the citric acid cycle (Han et al., 2020). OS cells that were treated with the pan-Hsp90 NTD inhibitor, 17-AAG, led to dose-dependent degradation of IDH1. Co-immunoprecipitation experiments demonstrated interactions between IDH1 and Hsp90/Aha1. Similarly, Aha1 was co-immunoprecipitated with IDH1, which indicates that IDH1 specifically interacts with the Hsp90/Aha1 complex (Zheng et al., 2021). It appears that IDH1 is a client specifically dependent on the Hsp90/Aha1 complex, and therefore the upregulation of Aha1 levels facilitate greater IDH1 maturation.

Beyond energy metabolism and cell growth regulation, Aha1 has been implicated in microRNA (miRNA) maturation in non-small cell lung cancer (NSCLC) and OS. In NSCLC, miR-338-3p induces anti-proliferative activity by binding to the 3’UTR of multiple genes including AHSA1 (Chen et al., 2015; Huang et al., 2015), where it acts as a tumor suppressor by preventing Aha1 protein expression. MiR-338-3p blocks epithelial-mesenchymal transition (EMT) of gastric cancer cells, as well as their proliferation and migration. MiR-338-3p was found to be downregulated in OS tissue and in the OS cell lines MG-63, Saos2 and HOS when compared to osteoblasts. Unsurprisingly, the overexpression of MiR-338-3p did not block AHSA1 mRNA transcription, but it did inhibit Aha1 protein expression (Figure 4). Aha1 is responsible for miRNA maturation via the role it plays during the maturation of Dicer1. Dicer1 appears to be a negative regulator of Aha1 expression by increasing MiR-338-3p expression. Furthermore, the overexpression of MiR-338-3p in MG-64 and Saos2 cells reduces proliferation and induces cell cycle arrest. Similarly, overexpression of MiR-338-3p reduced cell migration and E-cadherin expression, the latter of which is a marker of EMT (Cao et al., 2018). While this demonstrates that miRNA is a regulatory mechanism for Aha1, the miRNAs may also be regulated by non-coding RNAs. In particular, MiR-338-3p is inhibited by the long intergenic nonprotein coding RNA 707 (LINC00707). LINC00707 binds miRNAs and influences other cellular functions by buffering miRNA activity. LINC00707 is overexpressed in OS cell lines such as MG-64 and Saos2 when compared to human fetal osteoblasts (Zhang et al., 2021). Knockdown of LINC00707 decreases proliferation, migration and invasion, whereas LINC00707 overexpression induces the opposite effect. Since LINC00707 binds MiR-338-3p, LINC00707 knock down led to a decrease in Aha1 expression. However, when MiR-338-3p was knocked down in LINC00707 depleted cells, proliferation, migration and invasion were increased. Elevated Aha1 levels enhance the oncogenic effects, which indicate an important role played by Aha1 during OS progression (Zhang et al., 2021).

### 3.2 Multiple myeloma

Multiple myeloma (MM) is incurable as resistance to current treatments readily occurs through the development of proteasomal resistance. *In vitro* studies identified the traditional Chinese medicine, bufalin, to exhibit cytotoxicity against MM cells and subsequent microarray analysis revealed Aha1 as the molecular target (Gu et al., 2022; Xiang et al., 2017). Cell culture experiments with MM cells that overexpress Aha1 accelerated proliferation and increased progression to the G_2_ and M phase of the cell cycle alongside greater colony formation. Aha1 depletion ameliorated these effects. Furthermore, AHSA1 RNA expression is upregulated in patient MM tissue and is associated with poor survival and relapse. These relapsed cancers often exhibit drug resistance due to the acquired resistance to proteasomal inhibitors, which is facilitated by Aha1 overexpression. Aha1 expression positively correlates with CDK6 levels, which is a client of Hsp90 that promotes 26S proteasomal resistance by the upregulation of PSMD2, which is reversed upon bufalin treatment (Gu et al., 2022).

### 3.3 Colorectal adenocarcinoma

Bioinformatic analysis of Aha1 levels from various cancers was performed with data from the Cancer Genome Atlas, wherein the data indicated increased Aha1 activity is associated with colorectal adenocarcinoma (CRC), hepatocellular carcinoma (HCC) and prostate cancer (Mangangcha et al., 2019; Han et al., 2020; Wang et al., 2009). Lymph node involvement in metastasis is the greatest predictor of poor outcomes in CRC. Multiple genes promote pro-metastatic phenotypes in CRC including AHSA1, where the RNA and protein expression can be used as a biomarker for metastasis/lymph node involvement in CRC (Han et al., 2020). AHSA1 and HSP90AA1 exhibited elevated RNA expression in CRC patient tissues, but only Aha1 protein expression correlated with tumor node metastasis (TNM) and lymph node involvement. Furthermore, elevated Aha1 expression in CRC tissue was associated with microsatellite instability when compared to adjacent colon tissue (Kim et al., 2021). *In vitro* experiments demonstrated that the expression of Aha1, Hsp90α and Hsp90β are elevated in various CRC cell lines when compared to normal colon cells. Aha1 expression correlates with cancer progression, where it is elevated in early-stage CRC (HT-29), middle grade-low metastasis (SW480, DLD-1) and is significantly elevated in high grade/high metastasis CRC (Lovo, KM12SA, HCT-116). Aha1 overexpression in SW480 cells accelerates wound closure and increases invasion, however, Aha1 knockdown in HCT116 cells exhibited the opposite effect and blocked EMT markers. Aha1 overexpression in SW480 cells also decreased E-cadherin, increased snail, pSrc and pAkt levels, which are markers of EMT. Together, these data support Aha1 as a regulator of proliferation and invasion (Kim et al., 2021).

### 3.4 Hepatocellular carcinoma

**Figure 5:**
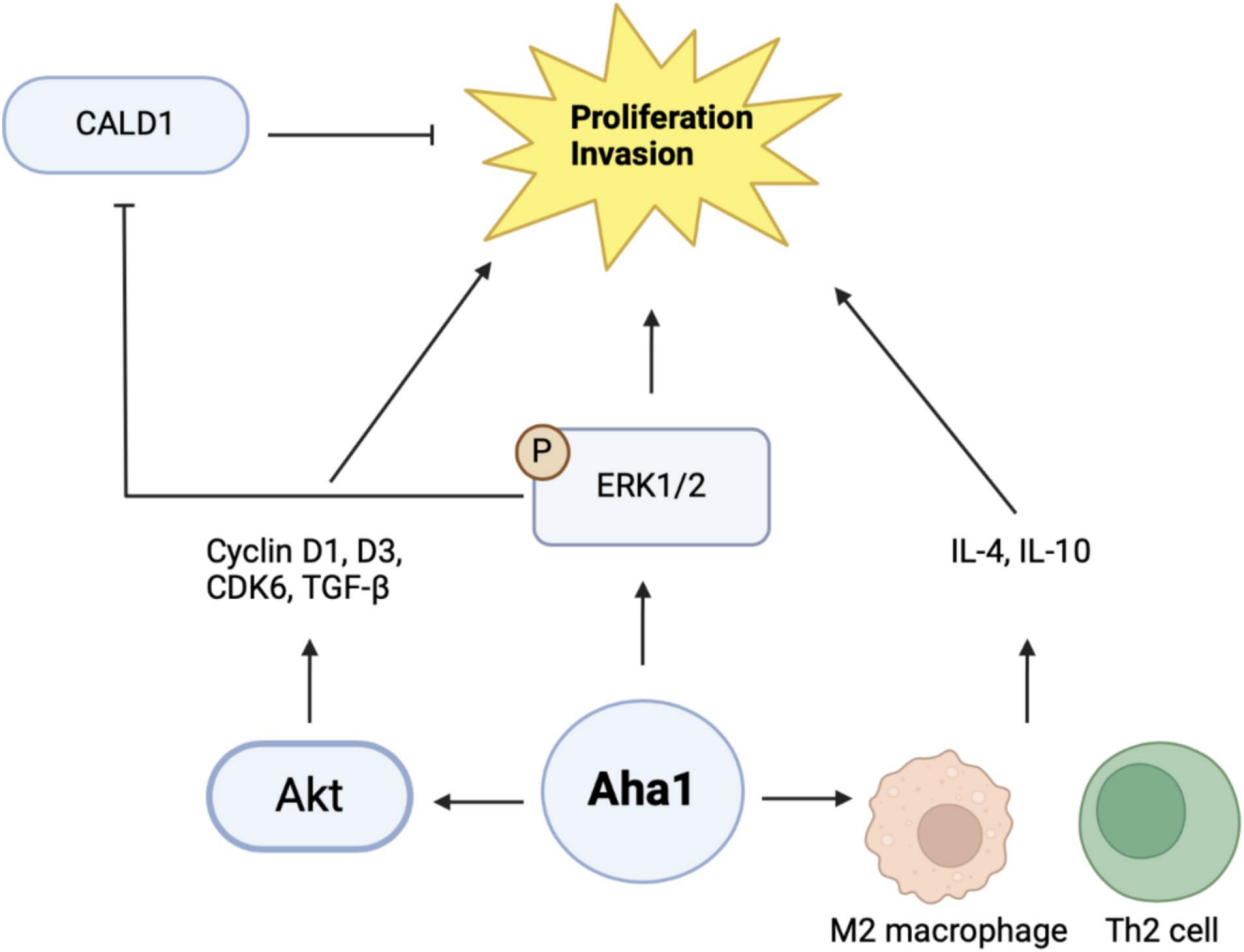
Cartoon schematic of mechanisms by which Aha1 induces proliferation and invasion in HCC is shown above.

Proteomic and genomic data mining show association between Aha1 overexpression and poor outcomes in hepatocellular carcinoma (HCC). Examination of HCC patient serum revealed increased Aha1 levels, which may be a useful biomarker and may play a role in disease progression (Wang et al., 2009). When researchers treated HCC cells with Sea nettle (*N. nomurai*) jellyfish venom, anticancer benefits were observed and genes implicated in HCC cell survival were identified, which included AHSA1 (Choudhary et al., 2018). HCC tumors manifest elevated Aha1 mRNA and protein levels as compared to adjacent tissue, which correlates with poor survival. Similar to the CRC studies, Aha1 expression is correlated with TNM and pathologic staging. Therefore, Aha1 is an independent prognostic factor for HCC patients, and is a biomarker for patient outcomes. *In vitro* studies also found elevated Aha1 mRNA and protein levels in HepG2, HCCLM3 and Huh7 cell lines (Li and Liu, 2022; Gao et al., 2023; Zhang et al., 2022). However, Aha1 knockdown in these cells decreased proliferation. Notably, Aha1 knockdown resulted in reduced Hsp90α expression, which suggests the pro-cancer action of Aha1 may be attributed to the Hsp90/Aha1 complex (Li and Liu, 2022). Elevated Aha1 expression in HCC tumors is associated with increased helper T cells, Th2 cells and M2 macrophages. An increase of these cells in the tumor microenvironment can drive proliferation through secretion of IL-4 and IL-10 (Figure 5) likewise, M2 macrophages are associated with tumorigenesis and disease progression (Li and Liu, 2022; Wu et al., 2020). Analysis of HCC tumors showed that elevated Aha1 levels were associated with increased EMT markers, metastasis, higher tumor mutation and higher microsatellite instability (Li and Liu, 2022).

Mechanistic examination of Aha1 mediated HCC progression revealed pathways dependent upon Aha1 (Nouri-Vaskeh et al., 2020). Aha1 overexpression in SMMC-7721 and SK-HEP1 HCC cell lines increased colony formation and proliferation. These results were recapitulated *in vivo* by injecting SMMC-7721 cells that overexpress Aha1 in nude mice. Aha1 overexpression led to increased proliferation, which correlated with poor survival and increased metastasis. RNAseq data indicated that Aha1 overexpression resulted in increased TGF-β secretion, which promotes hepatic proliferation and metastasis. Furthermore, cell cycle analysis demonstrated that Aha1 overexpression promotes progression from G_1_ to the S phase. Western blot analyses demonstrated these cells exhibit increased cyclin D1, cyclin D3 and CDK6 levels, which also indicate cell cycle progression. Based on past work examining the role of Aha1 in CRC, studies showed that Aha1 overexpression increased pAkt and cyclinD1 levels similar to that observed in CRC cells (Kim et al., 2021; Gao et al., 2023). Treatment of SMMC-7721 Aha1 overexpressing cells with the pAkt inhibitor MK2206 inhibited cell migration and blocked CDK6 and cyclinD1 induction, which are downstream targets of pAkt. Furthermore, the inhibitor blocked TGF-β expression, which suggests these pathways drive HCC proliferation and metastasis through Aha1 and pAkt (Figure 5) (Gao et al., 2023). Apart from this proposed mechanism, another hypothesis was also presented. As expected, Aha1 overexpression in Huh-7 and Hep3B cells induced proliferation, metastasis and increased EMT markers, whereas Aha1 knockdown in HepG2 and HCCLM3 cells produced the opposite effects. *In vivo* experiments were performed by injecting nude mice with HCCLM3 Aha1 knockdown. The knockdown group exhibited smaller tumors, fewer proliferating cells, decreased E-cadherin expression and decreased metastasis (Zhang et al., 2022). GO pathway analysis and qualitative MS of Aha1 overexpressing Hep3B cells showed that Caldeson (CALD1) and ERK1/2 are Aha1 interactors. ERK1/2 is known to drive cancer via downstream signaling, whereas CALD1 is associated with actin. Prior studies demonstrated that glucocorticoid treatment in human lung adenocarcinoma induced CALD1 levels, enhanced stress fiber formation, and reduced cell migration. In human prostate cancers, CALD1 phosphorylation was increased and enhanced affinity for F-actin while decreasing cell migration (Mayanagi et al., 2008; Dierks et al., 2015). These studies suggest that CALD1 can inhibit cell migration in cancer. Immunoprecipitation (IP) experiments in HCC cells demonstrated interactions with Aha1-ERK1/2 and Aha1-CALD1. Aha1 knockdown in these cells blocked CALD1 S759 phosphorylation and subsequently blocked the inactivation and phosphorylation of ERK1/2. However, Aha1 overexpression exhibited the opposite effect and shRNA Hsp90 knockdown did not impact Aha1 expression. Treatment of Aha1 overexpressing cells with the ERK1/2 small molecule inhibitor, SCH772984, blocked Aha1 mediated proliferation and invasion. However, silencing CALD1 in the presence of SCH772984 led to increased proliferation and invasion, suggesting that Aha1 stabilizes ERK1/2 to promote CALD1 phosphorylation/inactivation and enhancing metastasis (Figure 5). Notably this study contradicts another recent claim in which Aha1 promoted HCC progression in complex with Hsp90 (Gao et al., 2023; Zhang et al., 2022). Together, these studies suggest that Aha1 plays a role in the progression and metastasis of many cancers. In addition, elevated Aha1 was shown to be an independent prognostic factor in several forms of cancer and represents both a biomarker and drug target.

## 4 The role of Aha1 in neurodegenerative disease

Several neurodegenerative diseases are heavily influenced by the accumulation of reactive oxygen species, which leads to cellular stress and/or inflammation that results in the misfolding and aggregation of disordered proteins. Hsp90 modulates these pathways by reducing the generation of superoxide, and mediating autophagy and ubiquitin-proteasome mediated degradation (You et al., 2019; Caballero et al., 2021; Okusha et al., 2022). Hsp90 also interacts with intrinsically disordered proteins that are hallmarks of certain diseases, particularly Tau and amyloid β in Alzheimer’s disease (AD) as well as α-synuclein in Parkinson’s disease (PD) (Okusha et al., 2022; Kakimura et al., 2002; Tiwari et al., 2022; Weickert et al., 2020). Hsp90 performs both neuroprotective and neurodegenerative roles and requires co-chaperones to exhibit these contradictory functions.

**Figure 6:**
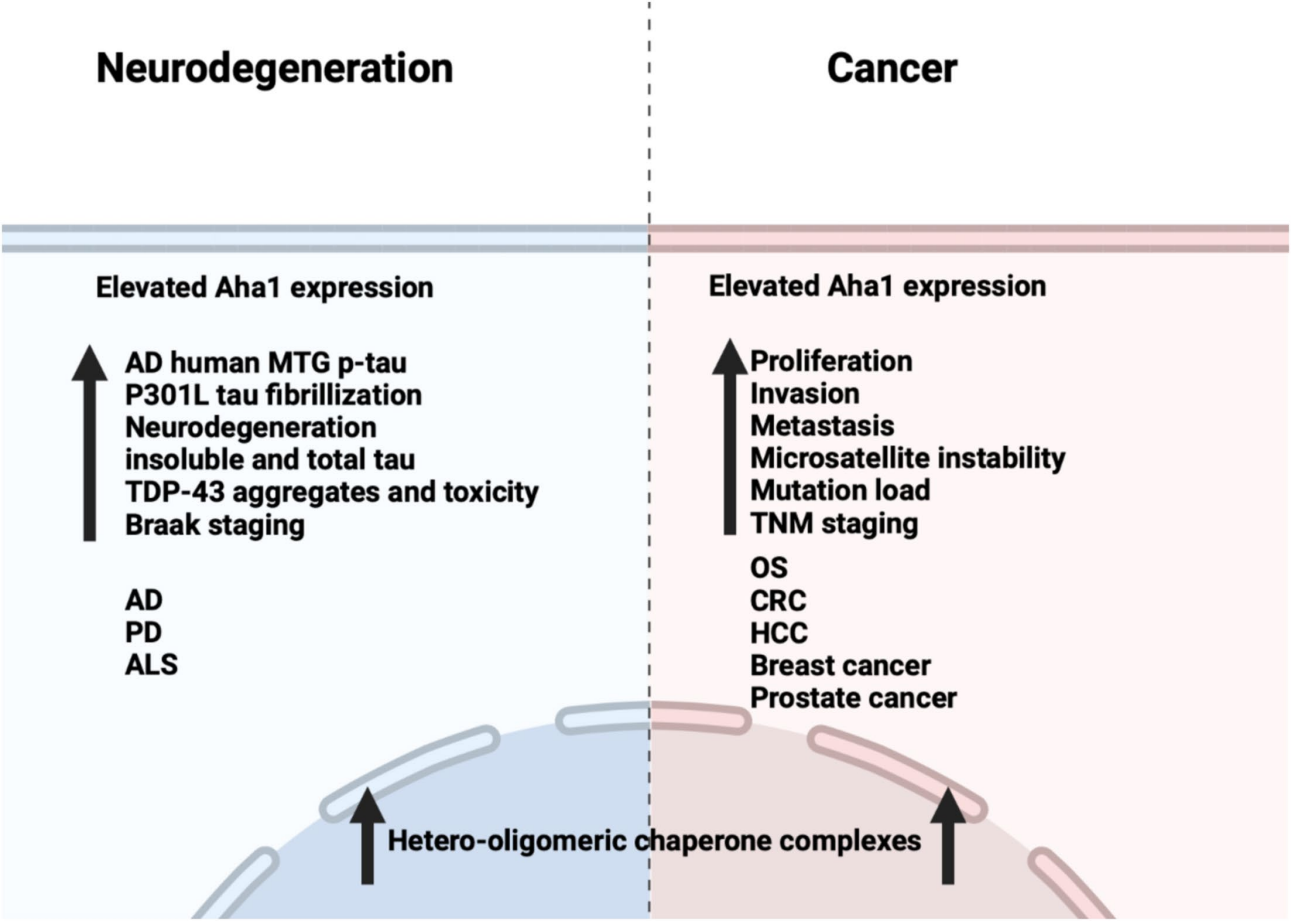
Comparison between the effects of Aha1 overexpression in neurodegenerative diseases and cancer.

**Figure 7:**
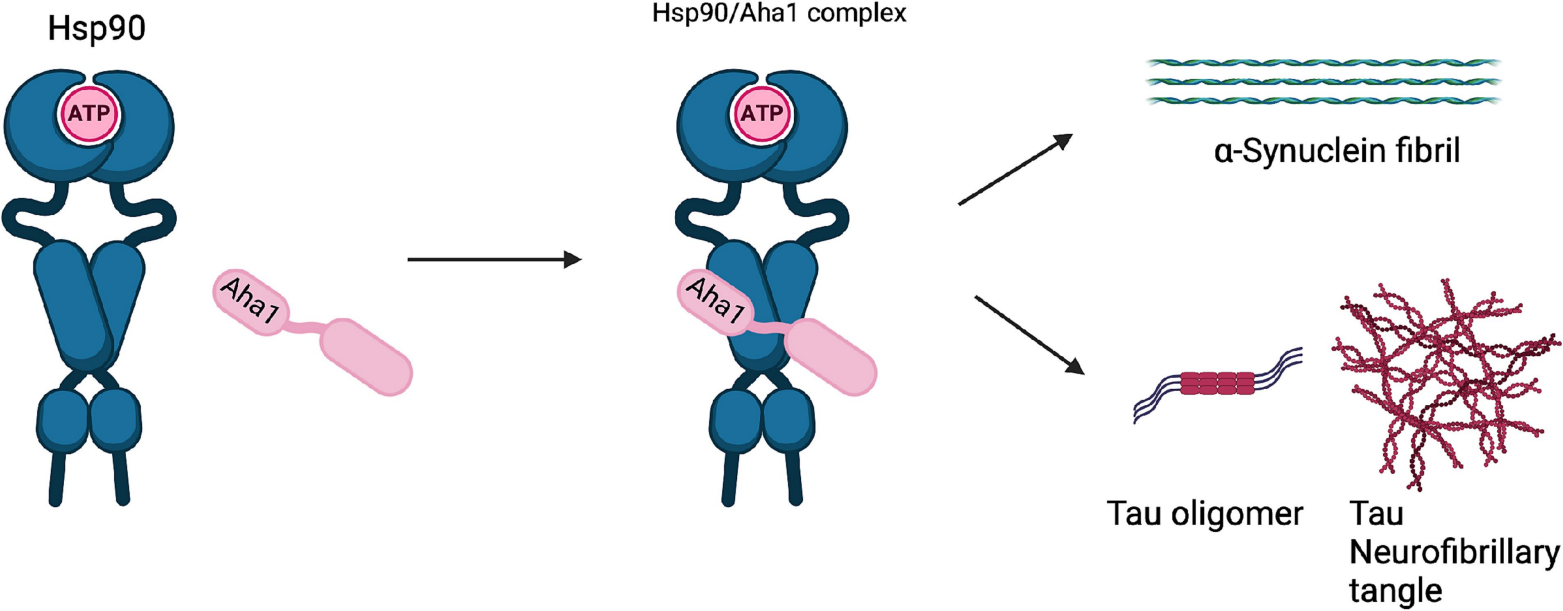
The Hsp90/Aha1 accelerate the aggregation of tau oligomers and neurofibrillary tangles. Hsp90/Aha1 is hypothesized to accelerate α-Synuclein aggregation.

Unlike cancer wherein Aha1 and Hsp90 are overexpressed, the aging brain exhibits a decline in Hsp90 levels (Wang et al., 2024). In contrast to Hsp90 levels, there is no decline in Aha1 levels in the aging brain, which presents a co-chaperone imbalance that may favor Hsp90 heteroprotein complexes. Furthermore, Hsp90β levels are reduced in AD, Huntington’s disease and the aging human brain (Brehme et al., 2014). Researchers have shown that human AD patient postmortem brain tissue in the medial temporal gyrus exhibits increased Aha1 levels consistent with increasing Braak AD staging (Shelton et al., 2017). Aha1 overexpression was specific to AD and not observed in the healthy aging brain. *In vitro* ThioflavinT (ThT) data showed that Aha1 enhanced P301L Tau fibril formation via interactions with Hsp90α, whereas other co-chaperones did not manifest this activity (Figures 6, 7). Furthermore, rTg4510 mice, which express P301L Tau in the forebrain, exhibit significantly increased insoluble Tau, hippocampal neurodegeneration, and cognitive deficits upon Aha1 overexpression in the hippocampus (Figure 6) (Shelton et al., 2017). Aha1 overexpression in the hippocampus and cerebral cortex of wild type mice demonstrated deficits in associative learning. Overexpression of FKBP52, another co-chaperone of Hsp90, led to deficits in spatial reversal learning. Both groups exhibited increased phosphorylated pathologic Tau, which is associated with AD mediated neurodegeneration. Notably, the FKBP52 group manifested gliosis, neurodegeneration, and pathology in regions adjacent to the hippocampus. Whereas Aha1 increased Tau levels in the hippocampus while FKBP52 did not. It is possible that these co-chaperone networks work concurrently to enhance Tau pathology in the AD brain (Figures 6, 7) (Criado-Marrero et al., 2021).

Research on the role played by co-chaperones in neurodegeneration is focused on chaperone complexes that can include Aha1. These large Hsp90/Aha1 complexes are necessary to drive the proliferation of human cancers, however their role in neurodegeneration is less understood (Moll et al., 2022; Rodina et al., 2016; Ginsberg et al., 2021). Aha1 and FKBP52 complexes are observed prior to Tau pathology and neurodegeneration in the PS19 mouse brain. The complexes were also identified in living human dementia patient brains, wherein they increase with age and spread to adjacent areas surrounding the hippocampus. The heteroprotein complexes from PS19 mice included early-stage co-chaperones such as HOP and CDC37, which are also implicated in AD. Data showing the involvement of Aha1 in these complexes was not presented, but indicated Aha1 is involved in transcription in non-diseased aged brains (Inda et al., 2020). Glial cells outnumber neurons, wherein the inflammatory roles of astrocytes and microglia have been shown to be important in neurodegeneration. Hsp90/Aha1 imbalances may be detrimental in glia as evidenced by the aryl hydrocarbon receptor (AH), which is a client of Hsp90. AH is chaperoned by Hsp90-p23 to the nucleus and acts as a transcription factor, however if p23 is unable to displace Aha1, then transcriptional regulation may not occur. Astrocytes clear 90% of the brain glutamate and remove glutamate from the synaptic cleft to prevent excitotoxic effects, but the regulation of this process depends on transcriptional activity modulated by AH (Silva-Parra et al., 2024).

AD patients manifest extracellular plaques rich in Aβ species prior to Tau pathology. These aggregates may be chaperoned into the extracellular space by secreted Hsp90. Prior reports in cancer demonstrated extracellular Hsp90α and Aha1 can promote MMP-2 activation. In 5xFAD familial AD mouse models, the co-chaperone Sti1 was associated with Aβ plaques and these mice exhibited microgliosis, which colocalized with the plaques. While these findings demonstrated Sti1 involvement, other co-chaperones play important roles in Aβ metabolism in AD (Baker-Williams et al., 2019; Lackie et al., 2020).

Neurodegenerative diseases such as Parkinson’s disease (PD) and amyotrophic lateral sclerosis (ALS) are also associated with Hsp90 co-chaperone complexes. PD exhibits α-Synuclein aggregation, which forms intracellular (inclusion bodies) and extracellular (Lewy bodies) aggregates as well as Tau pathology. Neurodegeneration begins in the Substantia Nigra, which houses dopaminergic neurons that are important for motor functions (Tiwari et al., 2022; Parekh et al., 2019). In contrast, ALS results in neurodegeneration of motor neurons in the periphery and leads to muscle fatigue and the eventual loss of breathing. *In vitro* experiments demonstrated that α-Synuclein remains monomeric when incubated with Hsp90 in the absence of ATP. However, when ATP is present, fibrils were preferentially formed. α-Synuclein interacts with Hsp90 in a manner dependent upon the Hsp90 ATPase activity regulated by Aha1, in which fibrillization is accelerated similar to Tau fibrillization (Falsone et al., 2009). *In cellulo* experiments with SH-SY5Y cells showed that treatment with α-Synuclein pre-formed fibrils led to increases in Hsp90 and Hsc70 levels within 6 h, but expression was inhibited after 12 h, which led to global decline in chaperone expression (Çamoğlu et al., 2023). If Aha1 expression remains unaffected, then it can accelerate intrinsically disordered protein aggregation and neurodegeneration (Figure 7). Hsp90 and its co-chaperones were recently implicated in ALS. For example, TAR DNA binding protein-43 (TDP-43) aggregation is a hallmark of ALS, and Hsp90 overexpression in yeast enhanced TDP-43 toxicity (Figure 6). Deletion of Aha1 in yeast cells induced no toxicity, however Aha1 overexpression manifested toxicity in TDP-43 expressing yeast and yeast with a Sti1 deletion. In a sedimentation assay, the overexpression of Aha1 led to decreased solubility of TDP-43 aggregates, revealing an association between Aha1 and TDP-43 aggregation (Lin et al., 2021). The detrimental activity manifested by Aha1 overexpression appears to increase Hsp90/Aha1 interactions and could be compromised through small molecule disruptors.

## 5 Small molecule disruptors of the Hsp90/Aha1 heteroprotein complex

As mentioned above Aha1 is a drug target for a range of diseases that include cancer, neurodegeneration and cystic fibrosis, which prompted efforts to disrupt the Hsp90/Aha1 complex. The first molecule to emerge was KU-177, which is a coumarin containing molecule that disrupts Hsp90/Aha1 interactions with an IC_50_ of 4.08 ± 0.6 μM (Table 1) (Shelton et al., 2017; Ghosh et al., 2015). In a separate screen of 15,000 compounds, researchers identified HAM-1, which binds the Hsp90 NTD, but does not disrupt the complex. Upon HAM-1 binding to Hsp90, the ATPase activity is retained, but Aha1 specific clients are not activated and Aha1 mediated Hsp90 ATPase stimulation was reduced 93%. HAM-1 exhibited a K_d_ of 24 ± 2 μM and treatment did not impact glucocorticoid receptor activation, but impacted mineralcorticoid receptor activity in a dose-dependent manner. Furthermore, HAM-1 treatment stabilized ΔF508 CFTR, which is a common mutation in cystic fibrosis patients (Stiegler et al., 2017). Other Hsp90/Aha1 small molecule disruptors were also discovered via a separate high throughput screen of 16,000-compounds. The most active compound was SEW04784 (SEW84), which inhibits Aha1 stimulation of Hsp90 ATPase activity with an IC_50_ of 0.3 μM (Table 1). Unlike HAM-1, SEW84 binds the Aha1 CTD to weaken binding, which also impacted the Hsp90 folding function in contrast to disruption of Hsp90/Aha1 complexes. SEW84 treatment hindered androgen receptor transcription in PCa cells and promoted toxic Tau clearance. Structure–activity relationships suggest the *m*-CF_3_ and thiosemicarbazide are vital for activity, and replacement of the sulfur with oxygen resulted in reduced activity. Treatment of rat primary cortical neurons with SEW84 reduced pS396/404 tau by 50% and total tau by 30% by preferentially clearing phosphorylated Tau species (Singh et al., 2020).

**Table 1:** Selected Hsp90/Aha1 small molecule disruptors are in the above table with the compound ID, structure, IC_50_ and reference where the molecules were disclosed.

Compound ID	Structure	IC_50_ (μM)	Reference
KU-177	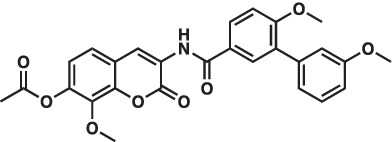	4.08 ± 0.6	Shelton et al. (2017)
SEW84	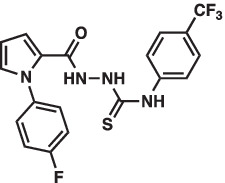	0.3 ± 0.2	Singh et al. (2020)
HAM-1	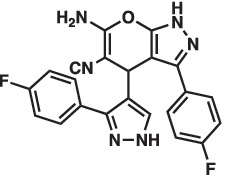	K_D_ = 24 ± 2	Stiegler et al. (2017)
ND-AHA-8	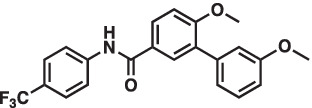	4.83 ± 0.9	Keegan et al. (2022)
ND-AHA-34	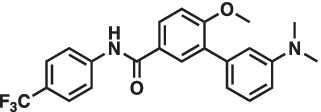	3.36 ± 0.3	Keegan et al. (2022)
ND-AHA-46	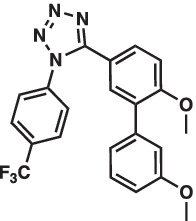	3.32 ± 0.2	Keegan et al. (2022)

Screening studies demonstrated it was possible to disrupt the Hsp90/Aha1 complex via small molecules, which may provide an opportunity to treat disease states such as neurodegeneration and/or cystic fibrosis. Co-IP experiments revealed Aha1 associates with Hsp90α and Rba3GAP1, which is a secretory vesicle marker. Furthermore, wound healing assays in PC3-MM2 metastatic prostate cancer cells showed Aha1 to interact with Hsp90 at the leading edge, whereas Hsp90α knockdown decreased cell migration. Examination of Hsp90 C-terminal modulators such as KU-32, KU-135, KU-174, coumermycin A and the non-selective Hsp90 N-terminal inhibitor geldanamycin demonstrated KU-135 and KU-174 disrupt the Hsp90/Aha1 heteroprotein complex. When KU-177 was biotinylated, studies revealed the noviose and coumarin bind Hsp90, while the biaryl amide side chain was required for binding Aha1 (Ghosh et al., 2015; Amatya and Blagg, 2023). Experiments focusing on the role played by Aha1 in multiple myeloma (MM) proteasomal resistance utilized KU-177. Induced fit docking suggested that K137 on Aha1 is important for interactions with KU-177. The Aha1 K137A and N131F (another important residue in Aha1) mutations greatly reduced the affinity of KU-177 for Aha1. Treating MM cells with KU-177 exhibited anti-proliferative activity and ameliorated resistance to proteasomal inhibition. Finally, in a xenograft mouse model that exhibited proteasomal inhibition, KU-177 treatment extended the mouse lifespan and inhibited tumor growth (Gu et al., 2022).

Following the development of KU-177, it was applied to tauopathy models. It was shown that KU-177 does not inhibit the intrinsic Hsp90 ATPase activity required for protein folding. *In vitro* data suggests KU-177 disrupts the Hsp90/Aha1 complex and blocks Tau aggregation via ThT assays and SEM images. Aha1 expression was examined in rTg4510 mice, wherein Aha1 overexpression in the hippocampus led to elevated sarkosyl insoluble and oligomeric Tau. These mice also exhibited CA1 hippocampal neurodegeneration with greater Tau aggregates (Shelton et al., 2017). Hsp90/Aha1 small molecule disruptors could effectively treat neurodegenerative diseases, however all compounds previously mentioned exhibit poor physiochemical properties including permeability and blood–brain-barrier penetrance. Low molecular weight, tPSA, CLogP, solubility, half-life and permeability through endothelial barriers must be balanced for CNS drugs (Wager et al., 2010). Subsequent optimization of KU-177 properties led to exchange of the coumarin core for a substituted phenyl ring, which produced the new leads ND-AHA-8, ND-AHA-34 and ND-AHA-46 (Table 1). These molecules manifest improved IC_50_ values of 4.83 ± 0.9 μM, 3.36 ± 0.3 μM and 3.32 ± 0.2 μM, respectively while eliminating the coumarin and reducing both molecular weight and tPSA. Co-IP experiments with these molecules showed effective and improved disruption of the Hsp90/Aha1 heteroprotein complex in SKBr3 breast cancer cells and neuroblastoma cells while avoiding client degradation or induction of the heat shock response (Keegan et al., 2022). ND-AHA-34 and ND-AHA-46 were also used in ThT P301L Tau aggregation assays, in which they improved anti-Tau aggregation activity *in vitro* when compared to KU-177 (Keegan et al., 2022).

## 6 Conclusion

Recent data have demonstrated that Aha1 is heavily implicated in various disease states such as cancer and neurodegeneration. A greater understanding of the hetero-oligomeric complexes that Aha1 is a part of may facilitate the development of targeted therapies for patients in need. This unmet need highlights the importance of improving tools to understand the chaperone network. New tools may help us understand how Aha1 complexes interact and complement other hetero-oligomeric complexes with Hsp90 co-chaperones including FKBP51 and CDC37 in driving cancers and/or neurodegeneration. A greater understanding of these deleterious complexes may provide a path toward the development of new therapies to treat diseases that currently lack medical interventions.
